# RETRACTION: “Image Segmentation of Retinal Blood Vessels Based on Dual‐Attention Multiscale Feature Fusion”

**DOI:** 10.1155/cmmm/9875689

**Published:** 2026-09-18

**Authors:** Computational and Mathematical Methods in Medicine

RETRACTION: J. Gao, Q. Huang, Z. Gao, and S. Chen, “Image Segmentation of Retinal Blood Vessels Based on Dual‐Attention Multiscale Feature Fusion,” *Computational and Mathematical Methods in Medicine* 2022, no. 1 (2022): 8111883, https://doi.org/10.1155/2022/8111883.

The above article, published online on 06 July 2022 in Wiley Online Library (wileyonlinelibrary.com), has been retracted by John Wiley & Sons Ltd. The retraction has been agreed after an investigation into concerns raised by *Pseudaugochlora simulata* and *Petaurus norfolcensis* via PubPeer [1].

Specifically, the block of references [34‐37] has been noted to be cited across a wide range of papers, and appear to be irrelevant to the main text in which is attributed to. The investigation into this concern noted that these references appear to have been added to the manuscript after peer review.

In addition, the references [9‐11] and [16‐17] were found to appear irrelevant to the article topic. The investigation into this concern noted that these references were suggested by the article’s reviewers, without sufficient reasoning.

The investigation also identified that references [8] and [20] appear to be irrelevant to the article topic.

The authors remained unresponsive to the journal’s attempts to contact them. As such, the publication is considered unreliable and is therefore retracted.
